# The Effect of Renal Function Impairment on the Mortality of Cirrhotic Patients: A Nationwide Population-Based 3-Year Follow-up Study

**DOI:** 10.1371/journal.pone.0162987

**Published:** 2016-09-15

**Authors:** Tsung-Hsing Hung, Chorng-Jang Lay, Chih-Wei Tseng, Chih-Chun Tsai, Chen-Chi Tsai

**Affiliations:** 1 Division of Gastroenterology, Department of Medicine, Dalin Tzu Chi Hospital, Buddhist Tzu Chi Medical Foundation, Chiayi, Taiwan; 2 School of Medicine, Tzu Chi University, Hualien, Taiwan; 3 Division of Infectious diseases, Department of Medicine, Dalin Tzu Chi Hospital, Buddhist Tzu Chi Medical Foundation, Chiayi, Taiwan; 4 Department of Mathematics, Tamkang University, Tamsui, Taiwan; Kaohsiung Medical University Chung Ho Memorial Hospital, TAIWAN

## Abstract

Renal function impairment (RFI) contributes to poor prognosis in cirrhotic patients. However, there have been no studies that seek to identify the effect of different types of RFI on the mortality of cirrhotic patients. We used the National Health Insurance Database, derived from the Taiwan National Health Insurance Program, to identify 44365 cirrhotic patients between January 1, 2007 and December 31, 2007. RFI was identified in 2832 cirrhotic patients, including 1075 with acute renal failure (ARF) (169 with hepatorenal syndrome, HRS; 906 with non-hepatorenal syndrome, NHRS), 705 with chronic kidney disease (CKD), and 1052 with end stage renal disease (ESRD). After Cox proportional hazard regression analysis adjusted by gender, age, and comorbid disorders, the 30-day, 30 to 90-day, 90-day to 1-year, and 1 to 3-year mortality hazard ratios (HR) compared to the non-RFI group were: (ARF) 5.19 (4.70–5.74), 3.23 (2.76–3.77), 1.51 (1.26–1.81), and 1.35 (1.13–1.61), respectively; (CKD) 2.70 (2.30–3.18), 2.03 (1.66–2.49), 1.60 (1.34–1.90), and 1.26 (1.06–1.49), respectively; and (ESRD) 1.42 (1.17–1.72), 1.62 (1.35–1.94), 1.90 (1.68–2.15), and 1.67 (1.48–1.89), respectively. Compared to NHRS, the 30-day, 30 to 90-day, 90-day to 1-year, and 1 to 3-year mortality HRs of HRS were 1.03 (0.80–1.32), 2.13 (1.46–3.11), 1.58 (0.90–2.75), and 2.51 (1.41–4.48), respectively, in cirrhotic patients with ARF. These results indicate the effects of CKD and ESRD on the mortality of cirrhotic patients are distributed equally in every survival stage, whereas the effect of ARF appears only in the early stage. Compared to NHRS, HRS contributes to a higher mortality risk at the late survival stage.

## Introduction

About 20% of inpatients with decompensated cirrhosis have renal function impairment (RFI) [1]. There are two forms of RFI, acute and chronic. Acute renal failure (ARF), a form of acute RFI, is attributed primarily to bacterial infection, gastrointestinal bleeding, or medication in cirrhotic patients. Hepatorenal syndrome (HRS), a special form of ARF in cirrhotic patients, is the ultimate result of arterial under-filling due to severe splanchnic and systemic vasodilatation [2]. According to the course of RFI, there are two types of HRS. Type 1 HRS progresses rapidly leading to renal failure in less than two weeks. Type 2 HRS is characterized by a steady increase in serum creatinine, frequently associated with refractory ascites [2]. Chronic kidney disease (CKD) and end stage renal disease (ESRD) are two chronic forms of RFI. Chronic RFI in cirrhotic patients is often attributed to diabetes mellitus, hypertension, glomerulonephritis, or nephrosclerosis [3]. Compared to CKD, patents with ESRD have little residual renal function and require maintenance dialysis for long-term vital organ replacement.

Recent studies have shown RFI is clearly associated with increased mortality, either in the intensive care unit or during hospitalization, and reduced 3- and 12-month survival in cirrhotic patients [4–6]. In our previous studies, we found that RFI contributed to a poor prognosis for cirrhotic patients with spontaneous bacterial peritonitis, and cirrhotic patients with ESRD had a better 3-year survival than those with CKD [7, 8]. However, it is still unknown if the different forms of RFI have the same effect on the long- and short-term survival of cirrhotic patients. In this study, we used a nationwide population-based database to enroll a large population of hospitalized cirrhotic patients with and without RFI. In addition, we divided mortality into four stages: 30-day, 30 to 90-day, 90 day to 1-year, and 1 to 3-year mortalities. The aim of this study was to identify the effect of different types of RFI on each mortality stage.

## Methods

### Ethical statement

This study was approval by the Institutional Review Board of the Buddhist Dalin Tzu Chi Hospital in Taiwan (B1010410). The review board waived the requirement for written informed consent from the patients involved, because all identifiable private information was stripped from the dataset released from the National Health Insurance Research Database (NHIRD).

### Database

The National Health Insurance Program started in 1995. The National Health Insurance Bureau (BNHI) now covers more than 99% of the Taiwanese population. All contracted medical institutions must provide medical records to the BNHI to receive medical payment. The BNHI employs professionals to review and screen the type, volume, quality and appropriateness of medical services provided by contracted institutions. These records for medical payment are established as a database, the NHIRD. Clinical researchers can apply for access to a dataset from the NHIRD for study, requiring the approval of the NHIRD research committee. The dataset used in this study was approved by the NHIRD research committee (agreement number 101516), including all International Classification of Diseases, 9th Revision, Clinical Modification (ICD-9-CM) codes of the inpatients in 2007, and excluded private information about patients or their health care providers.

### Study sample

We retrospectively enrolled inpatients having diagnostic codes for cirrhosis (ICD-9-CM code 571.5, or 571.2) between January 1, 2007 and December 31, 2007. Because the etiology of cirrhosis in young patients is different from adult patients, patients younger than 30 years of age were excluded. In addition, patients with biliary cirrhosis (ICD-9-CM code 571.6) and those receiving liver transplants were excluded. For those with multiple hospitalizations over this period, only the first episode was included. In total, 44365 cirrhotic patients were enrolled. Patients were divided into RFI- and non-RFI groups and were followed up individually for three years to identify their 3-year mortalities. Patients with RFI were defined as those with diagnostic codes related to RFI (ICD-9-CM code 584, 585, 586, 572.4, or other procedure codes relate to renal failure) [7].

RFI patients were stratified into the ESRD, ARF, and CKD groups according to a previous study [8]. ESRD patients were defined as those receiving long-term hemodialysis or peritoneal dialysis prior to admission. In Taiwan, patients with long-term dialysis receive certification to reduce their medical payment, and we were able to identify ESRD patients through the use of these certifications in their hospitalization. ARF was identified through the ICD-9-CM diagnosis code 584 and was divided into patients with NHRS and HRS. HRS patients were defined as those with the ICD-9-CM diagnostic code of 572.4, and NHRS patients were those with ARF but without HRS. With the exception of patients with ESRD, those with the diagnosis code of 585 were identified as patients with CKD. The ICD-9-CM diagnosis code of 586 is an obscure code for renal failure. We reviewed the other diagnosis and procedure codes for patients with the ICD-9-CM diagnosis code 585 to determine if they could be classified as ARF, CKD, or ESRD. In order to reduce the possibility of assigning the wrong code or classification, we reviewed all diagnoses and procedure codes for every hospitalization before enrolling patients. We used a national mortality database to match the selected patients to calculate their overall 30-day, 90-day, and 1-year mortality. The starting point was the date of admission in the enrolled hospitalization.

A number of mortality-related factors were selected as comorbid factors, including alcoholism (ICD-9-CM codes 291, 303, 305.00–305.03, 571.0–571.3), hepatic encephalopathy (ICD-9-CM code 572.2), hepatocellular carcinoma (ICD-9-CM code 155.0), diabetes mellitus (ICD-9-CM code 250), peptic ulcer bleeding (ICD-9-CM code: 531.0, 531.2, 531.4, 531.6, 532.0, 532.2, 532.4, 532.6, 533.0, 533.2, 533.4 and 533.6), esophageal variceal bleeding (ICD-9-CM code 456.0, 456.20), ascites (ICD-9-CM code 789.5, or procedure code 54.91), and bacterial infections. Bacterial infections included pneumonia (ICD-9-CM code 481–487, without 484), liver abscess (ICD-9-CM code 572.0), empyema (ICD-9-CM code 510), cellulitis (ICD-9-CM code 681 or 682), necrotizing fasciitis (ICD-9-CM code 728.86), central nerve system infection (including bacterial meningitis or brain abscess: ICD-9-CM code 324 or 320), septicemia (ICD-9-CM code 038, 020.0, 790.7, or 112.81), infective endocarditis (ICD-9-CM code 421), urinary tract infection (ICD-9-CM code 590.1, 595.0, 595.9 or 599.0), biliary tract infection or acute cholecystitis (ICD-9-CM code 576.1, 575.0, 574.00, 574.01, 574.30, 574.31, 574.60, 574.61, 574.80, 574.81), septic arthritis, (ICD-9-CM code 711), perianal abscess (ICD-9-CM code 566), and spontaneous bacterial peritonitis. Spontaneous bacterial peritonitis patients were defined as those with ICD-9-CM diagnosis codes 567.2, 567.8, or 567.9, without the procedure codes for abdominal surgery [9]. In addition to the above diagnosis codes, we checked all diagnosis codes of the enrolled patients to confirm those patients with bacterial infections.

### Statistical analysis

We used IBM SPSS Statistics package (IBM SPSS Statistics for Windows, Version 22.0. Armonk, NY: IBM Corp) to analyze data. The Chi square test or Fisher’s exact test were used to compare categorical variables. One-Way ANOVA was used to compare continuous variables. The Kaplan-Meier method was used to estimate the cumulative survival function from cirrhotic patients. In order to identify mortality-related factors, we used the proportional hazards Cox regression model to control for possible confounding factors. We presented hazard ratios (HR) with 95% confidence intervals (CI), and values of *P* < 0.05 were considered significant. The starting point for the evaluation of the 30-day, 90-day, 1-year, and 3-year mortalities was the date of admission in the enrolled hospitalization.

## Results

### Clinical characteristics and the mortalities of the cirrhotic patients with different types of RFI

A total of 44365 cirrhotic patients were enrolled in this study, including 2 832 (6.4%) with RFI, and 41533 (93.6%) without RFI. The mean age was 62.3 ± 13.1 years in RFI group and 59.4 ± 13.8 years in non-RFI group (*P* < 0.001). The demographic characteristics and comorbidities of the cirrhotic patients with and without RFI are shown in Table 1. In the RFI group, there were 1075 patients with ARF (169 with HRS and 906 with NHRS), 705 patients with CKD, and 1052 patients with ESRD. The 30-day, 90-day, 1-year, and 3-year mortalities were 8.0%, 16.3%, 32.8%, and 55.1%, respectively, in the non-RFI group, and 25.8%, 39.5%, 58.0%, and 76.3%, respectively, in the RFI group. Among patients with ARF, those with HRS had 30-day, 90-day, 1-year, and 3-year mortalities of 49.1%, 76.3%, 87.0%, and 95.9%, respectively, while those rates in the NHRS group were 42.3%, 55.6%, 66.9%, and 79.2%, respectively. The 30-day, 90-day, 1-year, and 3-year mortalities were 21.8%, 35.7%, 54.6%, and 73.2%, respectively, in the CKD group, and 10.5%, 22.3%, 48.0%, and 72.7%, respectively, in the ESRD group. These results are shown in Table 2.

**Table 1:** Demographic characteristics of cirrhotic patients with and without renal function impairment.

	RFI group (n = 2832)	Non-RFI group (n = 41533)	*P* value
Male, n (%)	1864 (65.8)	29551 (71.2)	< 0.001
Age, yrs	62.3 ± 13.1	59.4 ± 13.8	< 0.001
HCC, n (%)	620 (21.9)	13509 (32.5)	< 0.001
Hepatic encephalopathy, n (%)	401 (14.2)	4408 (10.6)	< 0.001
EVB, n (%)	217 (7.7)	4066 (9.8)	< 0.001
PUB, n (%)	165 (5.8)	2347 (5.7)	0.807
Alcoholism, n (%)	418 (14.8)	9545 (23.0)	< 0.001
Infection, n (%)	949 (33.5)	9317 (22.4)	< 0.001
Ascites, n (%)	694 (24.5)	7907 (19.0)	< 0.001
Diabetes mellitus	585 (20.7)	8034 (19.3)	0.087

RFI, renal function impairment; HCC, hepatocellular carcinoma; EVB, esophageal varices bleeding; PUB, peptic ulcer bleeding.

**Table 2:** The 30-day, 90-day, 1-year, and 3-year mortality of cirrhotic patients with different types of renal function impairment.

	Mortality			
	30-day (%)	90-day (%)	1-year (%)	3-year (%)
Non-RFI (N = 41533)	8.0	16.3	32.8	55.1
RFI^a^ (N = 2832)	25.8	39.5	58.0	76.3
ARF (n = 1075)	43.3	58.9	70.0	81.9
HRS (n = 169)	49.1	76.3	87.0	95.9
NHRS (n = 906)	42.3	55.6	66.9	79.2
CKD^b^ (n = 705)	21.8	35.7	54.6	73.2
ESRD^c^ (n = 1052)	10.5	22.3	48.0	72.7

^a^RFI includes ARF, ESRD, and CKD.

^b^CKD group: patients with chronic renal function impairment, not requiring dialysis before admission.

^c^ESRD group: patients requiring dialysis before admission. RFI, renal function impairment; ARF, acute renal failure; CKD, chronic kidney disease; ESRD, end stage renal disease; HRS, hepatorenal syndrome; NHRS, non-hepatorenal syndrome.

### The effects of different forms of RFI on the 30-day, 30 to 90-day, 90-day to 1-year, and 1 to 3-year mortalities

**Fig 1:**
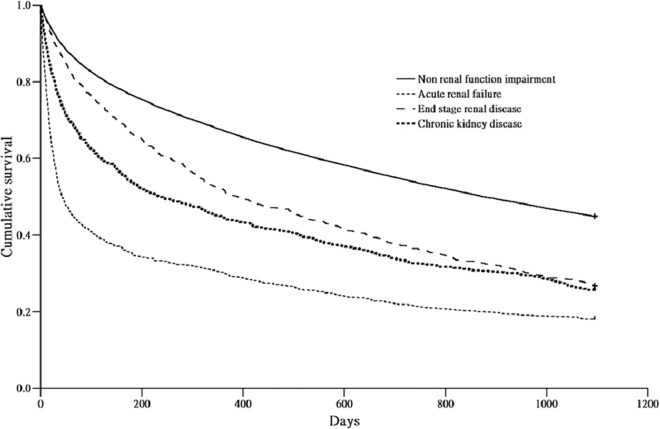
Cumulative survival plot for cirrhotic patients with acute renal failure, chronic kidney disease, and end stage renal disease, and without renal function impairment.

In order to evaluate the early and late effects of RFI on mortality, we calculated the 90-day mortality of the patients surviving more than 30 days, the 1-year mortality of the patients surviving more than 90 days, and 3-year mortality of the patients surviving more than 1 year. The 30 to 90-day, 90-day to 1-year, and 1 to 3-year mortalities were 9.0%, 19.7%, and 33.2%, respectively, in the non-RFI group, and 18.5%, 30.6%, and 43.6%, respectively, in the RFI group. The cumulative survival plot for cirrhotic patients with ARF, CKD, and ESRD, and without RFI is shown in Fig 1. After Cox proportional regression analysis adjusted by age, gender, and other comorbid factors, the HRs of RFI for 30-day, 30 to 90-day, 90-day to 1-year, and 1 to 3-year mortalities in cirrhotic patients were 3.26 (95% CI 3.01–3.54, *P* < 0.001), 2.19 (95% CI 1.97–2.44, *P* < 0.001), 1.71 (95% CI 1.56–1.87, *P* < 0.001), and 1.46 (95% CI 1.34–1.60, *P* < 0.001), respectively, compared to the non-RFI group. In cirrhotic patients with ARF, the HRs of the HRS group were 4.39 (95% CI 3.52–5.47, *P* < 0.001), 5.74 (95% CI 4.28–7.70, *P* < 0.001), 2.24 (95% CI 1.41–3.56, *P* < 0.001), and 2.60 (95% CI 1.57–4.12, *P* < 0.001), respectively, and HRs for the NHRS group were 5.32 (95% CI 4.77–5.92, *P* < 0.001), 2.77 (95% CI 2.31–3.33, *P* < 0.001), 1.43 (95% CI 1.17–1.74, *P* < 0.001), and 1.27 (95% CI 1.05–1.53, *P* = 0.010), respectively, for 30-day, 30 to 90-day, 90-day to 1-year, and 1 to 3-year mortalities, compared to the non-RFI group. The HRs for CKD patients were 2.70 (95% CI 2.30–3.18, *P* < 0.001), 2.03 (95% CI 1.66–2.49, *P* < 0.001), 1.60 (95% CI 1.34–1.90, *P* < 0.001), and 1.26 (95% CI 1.06–1.49, *P* = 0.004), and those for ESRD patients were 1.42 (95% CI 1.17–1.72, *P* < 0.001), 1.62 (95% CI 1.35–1.94, *P* < 0.001), 1.90 (95% CI 1.68–2.15, *P* < 0.001), and 1.67 (95% CI 1.48–1.89, *P* = 0.010) for 30-day, 30 to 90-day, 90-day to 1-year, and 1 to 3-year mortalities, respectively, compared to non-RFI group. These results are listed in Table 3.

**Table 3:** Adjusted hazard ratios of different types of renal function impairment for the 30-day, 30 to 90-day, 90-day to 1-year, and 1 to 3-year mortality of cirrhotic patients, compared with non-renal function impairment group.

Variables	Hazard ratio (95% confidence interval)			
	30-day	30 to 90-day	90 day to 1-year	1 to 3-year
RFI^a^	3.26 (3.01–3.54)	2.19 (1.97–2.44)	1.71 (1.56–1.87)	1.46 (1.34–1.60)
ARF	5.19 (4.70–5.74)	3.23 (2.76–3.77)	1.51 (1.26–1.81)	1.35 (1.13–1.61)
HRS	4.39 (3.52–5.47)	5.74 (4.28–7.70)	2.24 (1.41–3.56)	2.60 (1.57–4.12)
NHRS	5.32 (4.77–5.92)	2.77 (2.31–3.33)	1.43 (1.17–1.74)	1.27 (1.05–1.53)
CKD^b^	2.70 (2.30–3.18)	2.03 (1.66–2.49)	1.60 (1.34–1.90)	1.26 (1.06–1.49)
ESRD^c^	1.42 (1.17–1.72)	1.62 (1.35–1.94)	1.90 (1.68–2.15)	1.67 (1.48–1.89)

^a^RFI included ARF, ESRD, and CKD.

^b^CKD group: patients with chronic renal function impairment, not requiring dialysis before admission.

^c^ESRD group: patients requiring dialysis before admission. Hazard ratios were adjusted by patient gender, age, hepaotocellular carcinoma, hepatic encephalopathy, esophageal varices bleeding, peptic ulcer bleeding, alcoholism, and diabetes mellitus. RFI, renal function impairment; ARF, acute renal failure; HRS, hepatorenal syndrome; NHRS, non-hepatorenal syndrome; CKD, chronic kidney disease; ESRD, end stage renal disease.

### Different mortality effects of ARF and ESRD on the mortalities of cirrhotic patients

Compared to ESRD, the HRs for patients with ARF were 4.70 (95% CI 3.77–5.85, *P* < 0.001), 1.94 (95% CI 1.50–2.49, *P* < 0.001), 0.79 (95% CI 0.62–1.00, *P* = 0.048), and 0.92 (95% CI 0.73–1.17, *P* = 0.487) for 30-day, 30 to 90-day, 90-day to 1-year, and 1 to 3-year mortalities, respectively. The adjusted HRs for the HRS group were 5.86 (95% CI 3.42–6.90, *P* < 0.001), 4.64 (95% CI 3.08–7.00, *P* < 0.001), 1.62 (95% CI 0.96–2.73, *P* = 0.070), and 2.06 (95% CI 1.14–3.73, *P* = 0.016), and the HRs of the NHRS group were 4.57 (95% CI 3.66–5.72, *P* < 0.001), 1.62 (95% CI 1.23–2.12, *P* = 0.001), 0.73 (95% CI 0.57–0.93, *P* = 0.011), and 0.86 (95% CI 0.67–1.09, *P* = 0.215) for 30-day, 30 to 90-day, 90-day to 1-year, and 1 to 3-year mortalities, respectively, compared to ESRD. These results are presented in Table 4. The effects of HRS and NHRS on cirrhotic patient mortality are much different.

**Table 4:** Adjusted hazard ratios of acute renal failure for the 30-day, 30 to 90-day, 90-day to 1-year, and 1 to 3-year mortality of cirrhotic patients, compared to the end stage renal disease group.

Mortality	ARF (vs ESRD)		HRS (vs ESRD)		NHRS (vs ESRD)	
	HR (95% CI)	*p* value	HR (95% CI)	*p* value	HR (95% CI)	*p* value
30-day	4.70 (3.77–5.85)	< 0.001	5.86 (3.42–6.90)	< 0.001	4.57 (3.66–5.72)	< 0.001
30 to 90 day	1.94 (1.50–2.49)	< 0.001	4.64 (3.08–7.00)	< 0.001	1.62 (1.23–2.12)	0.001
90 day to 1 year	0.79 (0.62–1.00)	0.048	1.62 (0.96–2.73)	0.070	0.73 (0.57–0.93)	0.011
1 to 3 year	0.92 (0.73–1.17)	0.487	2.06 (1.14–3.73)	0.016	0.86 (0.67–1.09)	0.215

Hazard ratios were adjusted by patient’s gender, age, hepaotocellular carcinoma, hepatic encephalopathy, esophageal varices bleeding, peptic ulcer bleeding, alcoholism and diabetes mellitus. Abbreviations: HR = hazard ratios; CI = confidence interval; ARF = acute renal failure; HRS = hepatorenal syndrome; NHRS = non-hepatorenal syndrome; ESRD = end stage renal disease.

### Different mortality effects between HRS and NHRS on the mortality of cirrhotic patients

**Fig 2:**
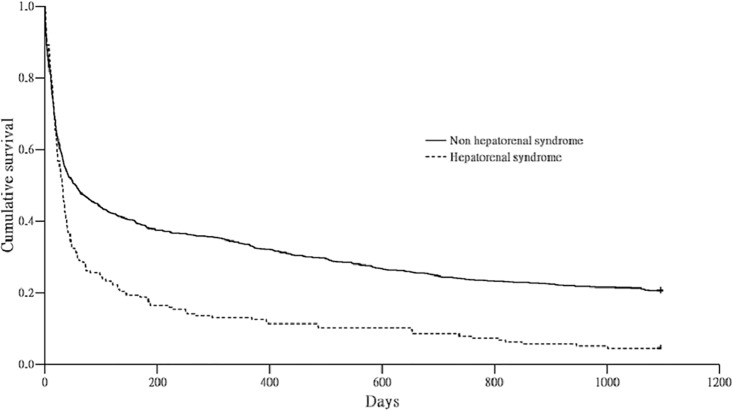
Cumulative survival plot for ARF cirrhotic patients with and without hepatorenal syndrome.

Compared to NHRS, the adjusted the HRs of the HRS group were 1.03 (95% CI 0.80–1.32, *P* = 0.844), 2.13 (95% CI 1.46–3.11, *P* < 0.001), 1.58 (95% CI 0.90–2.75, *P* = 0.110), and 2.51 (95% CI 1.41–4.48, *P* = 0.002) for 30-day, 30 to 90-day, 90-day to 1-year, and 1 to 3-year mortalities, respectively. The effects of NHRS and HRS on early mortality were the same, but HRS had a higher contribution to late-stage mortality. These results are shown in Table 5. The cumulative survival plot for ARF cirrhotic patients with and without HRS is shown in Fig 2.

**Table 5:** Adjusted hazard ratios of hepatorenal syndrome group for the 30-day, 30 to 90-day, 90-day to 1-year, and 1 to 3-year mortality of cirrhotic patients with acute renal failure, compared to non-hepatorenal syndrome group.

Mortality	HRS (vs. NHRS)	
	HR (95% CI)	*p* value
30-day	1.03 (0.80–1.32)	0.844
30 to 90 day	2.13 (1.46–3.11)	<0.001
90 day to 1 year	1.58 (0.90–2.75)	0.110
1 to 3 year	2.51 (1.41–4.48)	0.002

HR, Hazard ratio; CI, confidence interval; HRS, hepatorenal syndrome; NHRS, non-hepatorenal syndrome.

## Discussion

This is the first nationwide population-based cohort study to identify the effect of variable forms of RFI on mortality in cirrhotic patients. Due to the large population, this study provides reliable and important information about cirrhotic patients with RFI and may better reflect the real mortality in clinical practice. In addition, the results confirm the effect of variable forms of RFI on the different survival stages of cirrhotic patients.

Acute kidney injury (AKI) was introduced to replace ARF in 2004 because renal function changes can be trivial, and therefore should not be recognized as renal failure [10]. In a recent report, AKI was found in 47% of cirrhotic inpatients, consisting of 77% stage I, 11% stage II, and 12% stage III injuries [11]. In another study, AKI was found in 26% of cirrhotic inpatients, most of them stage I [12]. Stage III AKI was identified in only about 5% of cirrhotic inpatients. We used the ICD9 coding number to classify RFI cirrhotic patients, and AKI stages could not be identified exactly through this classification system. Based on the International Club of Ascites, ARF can be considered as AKIN stage III AKI [13]. The present study showed that 2.42% of cirrhotic inpatients in Taiwan could be classified as stage III AKI.

In a large population study, the proportion of decompensated cirrhotic patients with AKI increased from 1.5% in 2006 to 2.23% in 2012 [14]. The odds ratio of AKI for hospital mortality was 2.17 (95% CI 2.06–2.28, *P*<0.01). In another recent study, the prognosis of AKI was different between the patients with acute on chronic liver failure and those with acute decompensation of cirrhosis [15]. The present study showed that the mortality risk of ARF was greatest on the early survival stage in cirrhosis, and persisted at every survival stage for cirrhotic patients. The same as HRS, the mortality effect of NHRS persisted at all survival stage. This means that NHRS cannot be correct completely after treatment, and reflects the current concept that acute renal impairment can lead to permanent structural damage in those patients who survive the insult [16, 17].

Our results show that CKD and ESRD present similar high mortality risks for every survival stage in cirrhotic patients. That is reasonable because CKD and ESRD are persistent and uncorrectable conditions. In order to known the different mortality effect between ESRD and ARF, we performed another statistical comparison. We found that NHRS imposed a higher risk on short-term mortality, but a reduced risk on long-term mortality compared to ESRD, whereas HRS still imposed a higher risk on long-term mortality for cirrhotic patients. This result shows that the mortality risk of NHRS can be reduced to lower than ESRD after treatment. However, the mortality risk of HRS cannot be reduced to lower than ESRD. This reflects that the current treatment for HRS still is in a jam.

HRS is a functional type of renal failure in cirrhotic patients because it leads ultimately to splanchnic and systemic vasodilation. When the circulatory function is inadequate to restore hemodynamics, the release of vasoconstrictor mediators can result in severe renal vasoconstriction, causing renal failure [18]. Through liver transplantation, or pharmacological treatment with splanchnic vasoconstrictors and albumin, the condition is reversible [19, 20]. The prevalence of HRS increases with the progression of liver cirrhosis. HRS develops in 20% of cirrhotic patients with diuretic-resistant ascites [21]. The 3-month mortality of cirrhotic patients with HRS is approximately 85% [4]. Our results showed the incidence of HRS is only 0.38% of cirrhotic patients, and the 90-day mortality of HRS cirrhotic patients was 76.3%. The reduced mortality may be attributed to the progression of pharmacological treatment and prevention of HRS in recent years [22, 23]. However, the mortality of HRS cirrhotic patients was still very high.

Compared to NHRS, HRS imposed a much higher risk on 30- to 90-day, and 1- to 3-year mortalities. We consider that this is related to the common recurrence of HRS after discontinuation of treatment [19–23]. Current medical treatment apparently does not offer an effective means of maintaining survival in HRS patients. The uremic status in ESRD patients can be corrected by dialysis. However, dialysis is not predictive of improved transplant-free survival in cirrhotic patients with HRS [24]. MARS (the Molecular Adsorbent Recirculating System) and Prometheus (fractionated plasma separation and adsorption) have failed to decrease mortality in patients with HRS [25]. With the exception of liver transplantation, there is still no other therapy used to prolong long-term survival in cirrhotic patients with HRS.

There were several limitations to our study. First, the severity of liver cirrhosis could not be evaluated through the Mayo Clinic model for end-stage liver disease (MELD) score or the Child-Pugh score. This is because it was not possible to identify laboratory data such as bilirubin levels, albumin levels, or prothrombin time from ICD-9 coding numbers with the present database. Second, the exact etiology of non-alcoholic liver cirrhosis could not be identified. This is because the cause of cirrhosis is not always coded in their hospitalizations. However, previous studies have established the etiology of non-alcohol related cirrhosis in Taiwan is primarily related to the hepatitis B virus [26]. Third, comorbid disorders before 1995 could not be identified because the NHIRD only have data after 1995. However, because of the high mortality in complicated cirrhosis, we believe that the number of complicated patients before 1995 was very small in this study. Fourth, the severity of ARF or CKD could not be evaluated through the present database. Fifthly, we could not exactly identify the coexisting of CKD in the patient with ARF due to a lack of continuous laboratory data. Therefore, CKD patients with ARF were grouped into ARF group, not CKD group. Finally, HRS could be not stratified as type I or type II. This is the intrinsic limit on the database study.

Despite these limitations, this nationwide population-based study identified that the effects of CKD and ESRD on mortality for cirrhotic patients is distributed equally in every survival stage, unlike the effects of ARF, for which the effect is greatest over the first 90 days. Compared to NHRS, HRS has a higher impact on the 30- to 90-day, and 1- to 3-year mortalities in cirrhotic patients with ARF.
